# Determinants of Preeclampsia Among Pregnant Women in Public Hospitals of Wolaita and Dawuro Zones, Southern Ethiopia: A Case–Control Study

**DOI:** 10.1089/whr.2022.0043

**Published:** 2022-11-21

**Authors:** Abiyot Wolie Asres, Wakgari Binu Daga, Serawit Samuel, Getachew Asmare Adella, Shimelash Bitew Workie, Abinet Desalegn

**Affiliations:** ^1^ Department of Epidemiology and Biostatistics and School of Public Health, Wolaita Sodo University, Wolaita Sodo, Ethiopia.; ^2^ Department of Reproductive Health and Human Nutrition, School of Public Health, Wolaita Sodo University, Wolaita Sodo, Ethiopia.; ^3^ Department of Gynecology and Obstetrics, School of Medicine, Wolaita Sodo University, Wolaita Sodo, Ethiopia.

**Keywords:** preeclampsia, determinants, pregnant women, case–control, Ethiopia

## Abstract

**Introduction:**

Preeclampsia is a leading cause of maternal and fetal morbidity and mortality in Ethiopia. It is defined by the onset of new hypertension (HTN) and proteinuria in the second trimester of pregnancy. There is a research gap in the study area and there is an inconsistency of findings in previous studies. Therefore, this study aimed to determine the factors of preeclampsia among pregnant women in public hospitals.

**Methods and Materials:**

An institution-based unmatched case–control study was conducted in public hospitals in Wolaita and Dawuro Zones from February 1 to June 26, 2021. Women who were diagnosed with preeclampsia were cases, while those who did not have it were controls. They were selected using a consecutive sampling method. Descriptive statistics and logistic regression were done by STATA.

**Results:**

A total of 349 cases and 698 controls participated in this study. The average age of the cases and controls was 26.1 ± 4.6 standard deviation (SD) and 24.6 ± 4.8 SD years, respectively. The determinants of preeclampsia in this study were a family history of HTN (adjusted odds ratio [AOR = 11.5; 95% confidence interval, CI: 6.46–20.41], family history of diabetes mellitus [AOR = 2.1; 95% CI: 1.10–3.90], having two or multiple pregnancies [AOR = 6.33; 95% CI: 2.28–17.51], primigravida [AOR = 1.49; 95% CI: 1.01–2.21], and being gravida 5–9 [AOR = 2.47; 95% CI: 1.34–4.58]).

**Conclusion:**

In this study, family history of HTN, family history of diabetes mellitus, history of preeclampsia, primigravida, and multiple gestation pregnancies were the determinants of preeclampsia. As a result, health care providers should pay special attention to pregnant women with a family history of HTN, primigravida, and two or multiple gestation pregnancies during antenatal care follow-up.

## Introduction

Preeclampsia is a pregnancy complication that can develop after 20 weeks of pregnancy, during labor, or after delivery. Worldwide, preeclampsia and other conditions associated with pregnancy-related hypertension (HTN) are a major cause of sickness and death in both mothers and newborns. Preeclampsia is more prevalent among primigravidas, multigravidas, older mothers, and those with lengthy intervals between pregnancies, preexisting HTN or diabetes,^1–3^ multiple gestation pregnancies, obesity, and smokers.^4,5^

Preeclampsia is a substantial obstetrical problem in underdeveloped countries.^6^ Due to its unknown origin, preeclampsia continues to be the main cause of maternal and newborn infant death. About one-fourth of all medically complicated preterm deliveries are associated with preeclampsia.^7^ After obstetric hemorrhage, preeclampsia was the second most common reason for pregnancy-related critical care unit hospitalizations.^8^ Medical therapies are less effective in underdeveloped countries because of a lack of prenatal care and late diagnosis of complications. The problems do not manifest late, they are just detected and treated late. So the effects are more severe.^9^

Preeclampsia is associated with preterm birth, intrauterine growth restriction, intrauterine mortality, perinatal death, acute renal or hepatic failure, antepartum hemorrhage, postpartum hemorrhage, and maternal death,^10^ mothers' age, educational level, previously existing HTN, diabetes, primigravida, and obesity.^11^

Eighty-five percent of maternal deaths in Ethiopia are caused by direct obstetric complications (hemorrhage, obstructed labor, HTN during pregnancy, botched abortion, and infection). Because of this condition, perinatal mortality and morbidity among mothers and newborns in our nation have increased significantly.^12^ Therefore, this study aimed to determine the risk factors for preeclampsia among pregnant women attending antenatal care (ANC) or being admitted to public hospitals in the Wolaita and Dawuro Zones of Southern Ethiopia.

## Methods and Materials

### Study area and period

The study was conducted in the six public hospitals of Wolaita Sodo and Dawuro Zones in Southern Ethiopia from February 1 to June 26, 2021. The Southern Nation Nationalities and Peoples Regional State is 1 of 11 regions with a diverse range of nations and nationalities, each with its own unique culture, language, and way of life, weather, topography, habitats, and other natural occurrences. The Region is divided into 16 zones, with Wolaita Sodo and Dawuro Zones being included in the study because they are in the Wolaita Sodo University's catchment region. Wolaita Sodo is located 329 km south of Addis Ababa (Ethiopia's capital city) and 151 km southwest of Hawassa. Tercha Town, the administrative headquarters of the Dawuro zone, lies 500 km southwest of Addis Ababa and 275 km from Hawassa.

In Wolaita Sodo Zone, there are about six public and two private hospitals. Among the selected public hospitals, Wolaita Sodo University Comprehensive Specialized Teaching Hospital, Bodity, Bitena, Bale, and Bombe primary hospitals are found in Wolaita Sodo zone, whereas Tercha general hospital is found in Dawuro Zone. Wolaita Sodo University College of Health Science and Medicine is located in Wolaita Sodo town in 329 km south of Addis Ababa and 151 km from Hawassa.

These public hospitals provide services in the outpatient departments, emergency departments, gynecology and obstetrics wards, medical wards, pediatrics wards, and other rooms.

### Study design

An institution-based unmatched case–control study was conducted among pregnant women attending ANC and admitted for delivery in obstetrics and gynecology departments.

### Population

The source populations were all pregnant women who got ANC or delivery services in Wolaita Sodo and Dawuro Zones. The study population was pregnant women who came for ANC or delivery in the selected hospitals during the study period. All consecutively selected pregnant women and who came for ANC and were admitted for delivery in these hospitals were the study units.

### Case definitions

Cases are defined as pregnant women with preeclampsia who attended ANC or were admitted for delivery service in the selected hospitals. These were all pregnant women whose blood pressure was greater than or equal to 140/90 mmHg in two separate readings at least 4 hours apart and whose proteinuria was ≥300 mg per 24-hour urine collection or dipstick test reading ≥1+ after 20 weeks of gestation. They had been diagnosed and confirmed by obstetrics and gynecology physicians.

Controls are defined as those pregnant women without preeclampsia who came for ANC or were admitted for delivery service in these hospitals. This means pregnant women whose blood pressure was <140/90 mmHg and proteinuria <300 mg per 24 hours or <1+ on a urine dipstick test after 20 weeks of gestation in the same hospitals.

Cases and controls were identified through record review and after physician diagnosis during the study period in the ANC clinics and obstetrics and gynecology wards. The diagnosis includes history taking, clinical manifestations, and physical examination and laboratory tests.

Pregnant women who have eclampsia and preexisting renal disease, and are seriously ill, and whose gestational is <20 weeks were excluded through women's record review.

### Sample size determination

The sample size was calculated by using OpenEpi version 2.3 statistical software and the formula of two population proportions by assuming the case to control ratio 1:2, significant level 95% and power 80%. Among the factors taken from the previous study,^13^ age of women provided considerable sample size. Therefore, the proportion of age from 35 or more among women without preeclampsia was 0.55, whereas among cases was 0.45. The calculated sample size was 1065, of which 355 were cases and 710 controls.

### Sampling procedures

Due to the rare nature of the disease, the six public hospitals are selected purposively in Wolaita Sodo and Dawuro zones. The cases that fulfill the inclusion criteria were selected consecutively as they are diagnosed to have preeclampsia until the required sample size was attained. Then the next immediate two corresponding controls were selected consecutively on the same day and in the same ANC clinics and labour wards.

### Data collection procedures

The data were collected through record review, measurement and a face to face interview of pregnant woman using pretested questionnaires by a trained experienced health professional. The measurements included blood pressure, weight, and height and urine examination of the woman. They were interviewed about their sociodemographic characteristics, medical history, obstetric factors, and behavioral factors by trained health personnel immediately before and/or after ANC and delivery services.

A questionnaire was prepared by reviewing different literatures, including World Health Organization (WHO), Demography and Health Survey (DHS), family planning, and other documents, which are related to preeclampsia. Some questions were adopted from questionnaires used in other studies to investigate risk factors for preeclampsia. Then the designed questionnaire was changed from English to Amharic and back to English to check the consistency of the questionnaire.

Height and weight were measured with the participants wearing light clothing and no shoes. Weight of the woman was measured in kilogram and height will be measured in centimeter, while a woman was in standing position.

Blood pressure reading was taken while the woman was seated in the upright position using a mercury sphygmomanometer apparatus. For blood pressure, the participants were allowed to take rest for 5 minutes before taking blood pressure. The measurement was taken from participant's right hand, which covers two-thirds of the upper arm. Standard mercury sphygmomanometer was used throughout the study to minimize measurement error. Second blood pressure measurement was taken after 4 hours. If the second measurement becomes ≥140/90 mmHg, then she will be checked again after 4 hours to confirm the diagnosis.

Data regarding proteinuria and other clinical data were taken from the women's medical records or midstream urine sample was taken for each case and control if there was no previous record. Then proteinuria was assessed using urine dipstick method and part of the routine investigation for all pregnant women during the study period.

Thirty-four health personals (28 BSc, 6 MPH specialty health professionals) were recruited as data collector and supervisor, respectively, in these hospitals.

### Data quality management

Data collectors and supervisors received training roughly 10 days before data collection. At the start of the interview, the respondents were given a clear description of the study's purpose and objective. Before 1 week of actual data collection, a pretest was conducted from other hospital respondents other than the selected one to check consistency and any ambiguity in the language after switching from English to Amharic and local languages. Some questionnaires were changed as a result of the pretest. Throughout the data gathering methods, the principal investigator and recruited supervisors kept a tight supervision on the collected data. The data collectors and supervisors double-checked each respondent's data for completeness, clarity, consistency, and accuracy.

### Data processing and analysis

The data were cleaned, coded, and entered into EpiData version 4.6 software once collected. The data were then translated into STATA Version 14 and analyzed. There were *post hoc* tests and descriptive analyses performed. Bivariate logistic regression was used to examine the association between preeclampsia and each variable, and variables with a *p*-value <0.20 were included in the final multiple logistic regression models. Variables with a *p*-value <0.05 in the multivariable logistic regression model were considered statistically significant factors. The findings were presented using text and tables.

### Ethical consideration

An official approval letter was obtained from Wolaita Sodo University Institutional Review Board. Another official support letter was obtained from each selected hospital administration office. Finally, an informed consent was obtained from each respondent after explaining the purpose of the study. Confidentiality and privacy of the respondents' responses were maintained during data collection, analysis, and reporting of findings using computer password.

## Results

### Sociodemographic characteristics of the respondents

A total of 349 cases and 698 controls took part in this investigation. The average age of the case and control groups was 26.1 ± 4.6 years and 24.6 ± 4.8 years, respectively. In terms of educational status, around 50 (14.3%) cases and 76 (10.9%) controls are unable to read and write (Table 1).

**Table 1.** Sociodemographic Characteristics of Women who Attended Antenatal Care and Delivery Service in Hospitals

Sociodemographic characteristics	Case (349), ***n*** (%)	Control (698), ***n*** (%)
Mother's age (years)		
<20	48 (13.7)	155 (22.2)
20–29	217 (62.2)	431 (61.8)
30–39	84 (24.1)	108 (15.5)
40–49	0 (0.0)	4 (0.5)
Mother's education		
Cannot read and write	50 (14.3)	76 (10.9)
Can read and write only	80 (22.9)	149 (21.3)
Primary school	40 (11.5)	78 (11.2)
Secondary school	74 (21.2)	175 (25.1)
Diploma and above	105 (30.1)	220 (31.5)
Occupation		
Housewife	173 (49.6)	288 (41.4)
Merchant	46 (13.2)	88 (12.6)
Government employee	95 (27.2)	163 (23.4)
Student	31 (8.9)	143 (20.6)
Other^a^	4 (1.2)	14 (2.0)
Current residence		
Urban	217 (62.2)	476 (68.4)
Rural	132 (37.8)	220 (31.6)
Median monthly income		
<4750 Birr	121 (49.6)	254 (51.8)
≥4750 Birr	123 (50.4)	236 (48.2)

^a^ Other: Student laborer, hotel, café and restaurant worker.

### Medical factors of preeclampsia

About 78 (22.4%) cases and 24 (3.4%) controls, and 47 (13.5%) cases and 21 (3.0%) controls had family history of HTN and preeclampsia, respectively (Table 2).

**Table 2.** Distribution of Medical Illness Factors of Women Attending Antenatal Care and Delivery

Medial illnesses	Response category	Case (349), ***n*** (%)	Control (698), ***n*** (%)
Family history of HTN	Yes	78 (22.4)	24 (3.4)
	No	271 (77.6)	674 (96.6)
Family history of preeclampsia	Yes	47 (13.5)	21 (3.0)
	No	302 (86.5)	677 (97.0)
Family history of DM	Yes	67 (19.2)	35 (5.0)
	No	282 (80.8)	663 (95.0)
History of miscarriage/abortion	Yes	67 (19.2)	88 (12.6)
	No	282 (80.8)	608 (87.4)
No. of miscarriage/abortion	1	45 (67.2)	66 (75.0)
	≥2	22 (32.8)	22 (25.0)
Maternal history of preeclampsia	Yes	91 (26.1)	5 (0.7)
	No	258 (73.9)	693 (99.3)
Maternal history of renal disease	Yes	46 (8.2)	65 (9.3)
	No	303 (86.8)	633 (90.7)

DM, diabetes mellitus; HTN, hypertension.

### Obstetrics factors

Before their current pregnancy, 213 (61.0%) of cases and 420 (60.5%) of controls were using contraception. Among the majority of women who visited ANC or delivered in these hospitals, 328 (94.5%) cases and 668 (95.7%) controls had a history of ANC follow-up, 298 (85.4%) cases and 640 (92.4%) controls were gravida 1, and 285 (90.8%) cases and 598 (95.7%) controls were gravida 2. Similarly, 14 (4.0%) cases and 13 (1.9%) controls had twin or multiple gestation pregnancies (Table 3).

**Table 3.** Obstetrics Factors of Women who Attended Antenatal Care and Deliver Service in the Hospitals, Ethiopia

Obstetric factors	Response category	Case, ***n*** (%)	Control, ***n*** (%)
Contraceptive use	Yes	213 (61.0)	420 (60.5)
	No	136 (39.0)	274 (39.5)
Gravidity	Gravida 1	123 (35.2)	288 (41.6)
	Gravida 2–4	175 (50.1)	352 (50.8)
	Gravida ≥5	51 (14.6)	53 (7.7)
Parity	Nulliparous	33 (10.5)	93 (14.9)
	Parous	281 (89.5)	532 (85.1)
Multiplicity of current pregnancy	Single	335 (96.0)	685 (98.1)
	Multiple	14 (4.0)	13 (1.9)
Sex of the fetus	Male	102 (29.2)	219 (31.4)
	Female	104 (29.8)	254 (36.4)
	Did not known	143 (41.0)	225 (32.2)
Ever attended ANC	Yes	328 (94.0)	668 (95.7)
	No	21 (6.0)	30 (4.3)

ANC, antenatal care.

### Bivariate and multivariate logistic regression result of the study

To evaluate the related determinants of preeclampsia in the study area, we used multivariate logistic regression analysis. Variables with a *p*-value <0.20 were considered eligible for multivariate logistic regression analysis, and variables with a *p*-value <0.05 were regarded substantially associated factors of preeclampsia in the multivariable logistic analysis. The women's educational status, family history of HTN, family history of diabetes mellitus (DM), maternal history of HTN, history of DM, history of preeclampsia, history of abortion, history of urinary tube infection, multiplicity of pregnancy, parity, and gravidity were all eligible for multivariate logistic regression analysis.

In the multivariate analysis, six of these characteristics were associated with preeclampsia. They include HTN in the family, family history of DM, history of HTN, maternal history of preeclampsia, multiplicity of pregnancies, and gravidity.

When compared to controls, pregnant women with a family history of HTN were 7.2 (3.73–13.94) times more likely to develop preeclampsia. Pregnant women with a history of preeclampsia were 49.5 times more likely to develop preeclampsia than women without a history of preeclampsia. Furthermore, compared to singleton pregnancy, pregnant women who had two and multiple gestation pregnancies were 6.41 (2.00–20.54) times more likely to have preeclampsia (Table 4).

**Table 4.** Determinants of Preeclampsia Among Women who Attended Antenatal Care and Delivery in Hospitals

Characteristics	Response category	Case	Control	95% CI	
		No. (%)	No. (%)	COR 95% CI	AOR 95% CI
Educational status	Illiterate	50 (14.3)	76 (10.9)	1.37 (0.9–2.0)	1.0 (0.6–1.7)
	Read and write	299 (85.7)	622 (89.1)	1	1
Family history of HTN	Yes	78 (22.4)	24 (3.4)	8.1 (5.0–13.0)	7.2 (3.7–13.9)
	No	271 (77.6)	674 (96.6)	1	1
Family history of DM	Yes	67 (19.2)	35 (5.0)	4.50 (2.9–6.9)	2.1 (1.1–3.9)
	No	282 (80.8)	663 (95.0)	1	1
History of HTN	Yes	47 (13.5)	1 (0.1)	108 (14.9–79.8)	41.5 (5.3–32.8)
	No	302 (86.5)	697 (99.9)	1	1
History of miscarriage/abortion	Yes	67 (19.2)	88 (12.6)	1.64 (1.2–2.3)	1.0 (0.6–1.7)
	No	282 (80.8)	608 (87.4)	1	1
Urinary tract infection	Yes	46 (13.2)	65 (9.3)	1.5 (0.9–2.2)	0.8 (0.4–1.4)
	No	303 (86.8)	633 (90.7)	1	1
Maternal history of preeclampsia	Yes	91 (26.1)	5 (0.7)	48.9 (19.6–60.6)	49.5 (16.7–70.9)
	No	258 (73.9)	693 (99.3)	1	1
Parity (939)	Nulliparous	33 (10.5)	93 (14.9)	0.67 (0.4–1.0)	0.64 (0.4–1.1)
	Parous	281 (89.5)	532 (85.1)	1	1
Gravidity	Gravida 1	123 (35.2)	288 (41.6)	0.86 (0.6–1.1)	1.64 (1.1–2.5)
	Gravida 2–4	175 (50.1)	352 (50.8)	1	1
	Gravida ≥5	51 (14.7)	53 (7.6)	1.94 (1.3–2.9)	1.61 (0.9–2.9)
Multiplicity of pregnancy	Single	335 (96.0)	685 (98.1)	1	1
	Multiple	14 (4.0)	13 (1.9)	2.20 (1.02–4.7)	6.41 (2.0–20.5)

AOR, adjusted odds ratio; CI, confidence interval; COR, crude odd ratio.

## Discussion

The aim of this study was to find out what factors contributed to preeclampsia in pregnant women who went to ANC or were hospitalized to public hospitals in the Wolaita and Dawuro zones. According to our data, family history of HTN, family history of DM, history of preeclampsia, having a twin or multiple gestation pregnancies, and gravidity were determinants of preeclampsia.

A family history of HTN has been associated to preeclampsia. Cases with a family history of HTN were 7.2 times more likely to develop preeclampsia than controls. This is in line with the previous study.^14–17^ The reason may be explained as HTN has high familial tendency to be transferred genetically to their children. This is due to families' sharing main things in their life like wealth, sedentary life, nutrition, working condition, and others.

Furthermore, a family history of diabetes was a significant risk factor for the development of preeclampsia. Pregnant women with a history of DM were more prone to developing preeclampsia compared with women without preeclampsia. The finding was comparable with studies done in Ethiopia.^18^ This may be due to the fact that high levels of plasma triglycerides cause endothelial cells to accumulate triglycerides in diabetic women, resulting in endothelial cell dysfunction and a predisposition to high blood pressure.^19^

Maternal history of preeclampsia was one of the determinants of preeclampsia. This finding is in line with a study conducted in Sweden.^20^ According to a study conducted in Sweden, preeclampsia in one pregnancy is a poor predictor of subsequent pregnancy, but it is a good predictor of preeclampsia in subsequent pregnancies. The risk of preeclampsia in multiparous women was 4.1% in the first pregnancy and 1.7% in later ones. The risk was lower for women without a history of the condition in their first pregnancy or those who had one or two previous pregnancies. The proportion of women who went on to have a further pregnancy was 4%–5% lower after having a pregnancy with any preeclampsia, but over 10% lower if it was associated with very preterm delivery.

In the research area, primigravida and multiple pregnancies were also found to be independent factors of preeclampsia. This is in line with a study conducted previously.^14–17^ Even though the exact mechanism of this is unknown, it is assumed to be linked to decreased placental perfusion, which causes systemic vascular endothelial dysfunction.^21,22^ This increase is attributable to a less successful cytotrophoblastic invasion of the uterine spiral arteries. The resulting placental hypoxia triggers a cascade of inflammatory events, altering the balance of angiogenic factors and triggering platelet aggregation, leading to endothelial dysfunction and the preeclampsia syndrome. Women who have been pregnant for the first time might not adapt to the physiological changes of pregnancy. Here, early in pregnancy, new blood vessels develop and evolve to efficiently send blood to the placenta. On the other hand, twin pregnancies may be due to increased placental mass that leads to increased circulating levels.^23^

The mother's age and pregnancy with a new partner were previously connected to preeclampsia, despite the fact that they were not mentioned in this study.^16,24^ In contrast to other studies,^25^ maternal age >35 years was not a risk factor in this study. The reason for this could be that, due to recall bias, women in our society may not know their exact age or birth date.

## Conclusion

In this study, we identified positively and negatively associated factors with preeclampsia. Family history of HTN, family history of DM, maternal history of preeclampsia, being primigravida, and having multiple pregnancies were risk factors for preeclampsia. As a result, health care providers should pay special attention to pregnant women with a family history of HTN, primigravida, and twins or more pregnancies during ANC follow-up. Encouraging pregnant women's health-seeking behavior would provide a chance to diagnose preeclampsia as early as possible.

## Abbreviations Used

ANC: antenatal care
AOR: adjusted odds ratio
CI: confidence interval
COR: crude odd ratio
DM: diabetes mellitus
HTN: hypertension
SD: standard deviation
